# Foodborne botulism outbreak involving different nationalities during the Rugby World Cup: critical role of credit card data and rapid international cooperation, France, September 2023

**DOI:** 10.2807/1560-7917.ES.2023.28.47.2300624

**Published:** 2023-11-23

**Authors:** Laure Meurice, Laurent Filleul, Aurélie Fischer, Annie Burbaud, Gauthier Delvallez, Laure Diancourt, Sophie Belichon, Benjamin Clouzeau, Denis Malvy, Magali Oliva-Labadie, Coralie Bragança, Hendrik Wilking, Rafaela Franca, Greg Martin, Gauri Godbole, Mathieu Tourdjman, Nathalie Jourdan-Da Silva

**Affiliations:** 1 Santé publique France, Regional office in Nouvelle-Aquitaine, Bordeaux, France; 2 Regional health agency Nouvelle-Aquitaine (ARS Nouvelle-Aquitaine), Bordeaux, France; 3 National Reference Center for Anaerobic Bacteria and Botulism, Institut Pasteur, Université Paris Cité, Paris, France; 4 Direction Générale de l'Alimentation (DGAL), Paris, France; 5 Bordeaux Hospital Center, Bordeaux, France; 6 Poison control center, Bordeaux Hospital Center, Bordeaux, France; 7 Robert Koch Institute, Department of Infectious Disease Epidemiology, Berlin, Germany; 8 Health Service Executive - Health Protection Surveillance Centre, Dublin, Ireland; 9 United Kingdom Health Security Agency, Gastrointestinal Pathogens and Food Safety (One Health) Division, London, United Kingdom; 10 Santé publique France, Department of Infectious Disease, Saint-Maurice, France

**Keywords:** botulism, Clostridium botulinum, foodborne diseases, disease outbreak, mass gathering, credit card data

## Abstract

In September 2023, a severe outbreak of type B botulism with fifteen cases was linked to consumption of canned sardines at a restaurant in Bordeaux, France, during the Rugby World Cup. The cases were from seven countries. One death was recorded. Outbreak investigation using credit card data, rapid communication between health authorities of the affected countries and broad media communication allowed identification of cases and exposed persons and prevented further severe outcomes.

An unprecedented outbreak of 15 cases (including one death) of foodborne botulism occurred in Bordeaux, France, in September 2023 during the Rugby World Cup. Here we describe the national and international outbreak investigation using credit card data and control measures taken.

## Outbreak detection and investigations

On 10 September 2023, the Bordeaux University Hospital reported three suspected cases of botulism to the local public health authorities [1]. All suspected cases had visited the same restaurant (Restaurant A) in Bordeaux on different dates and reported consumption of canned marinated sardines. The sardines were part of a batch made by Restaurant A on 1 September 2023 and served between 1 and 10 September. Cases were of different nationalities. In the previous days, the city had hosted two international rugby games as part of the Rugby World Cup attended by a large number of international visitors. An investigation was initiated to identify and contact persons visiting restaurant A and to contact public health agencies of countries whose citizens were affected by the outbreak.

Considering the severity of botulism and the urgency of control measures to stop the outbreak, an active search for persons who had consumed the sardines was performed by using data retrieved from credit card receipts of restaurant A.

A suspected case of botulism was defined as a person with symptoms compatible with botulism (oculomotor palsy, mydriasis, ptosis, dysphagia, nausea, vomiting, diarrhoea), living or visiting the Bordeaux area and visiting Restaurant A between 1 and 10 September 2023. A confirmed case was defined as detection of type B botulinum neurotoxins (BoNT) in stool and/or serum samples and/or detection of type B *C. botulinum* in stools from a suspected case.

**Figure 1:**
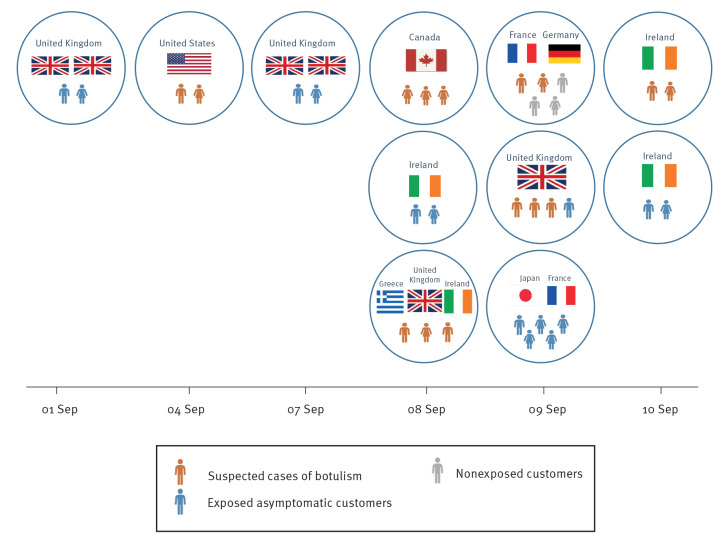
Identification of persons visiting and ordering or consuming canned sardines at Restaurant A, Bordeaux, France, 1–10 September 2023 (n = 32)

By screening meal orders and credit card receipts of Restaurant A, we identified 29 customers who had ordered canned sardines (Figure 1). Among these, 12 had already been identified as suspected cases, 14 were contacted by the French or British health authorities and were considered non-cases as they did not present any symptoms and three were symptomatic British citizens who were urgently referred to an emergency care in the United Kingdom (UK) on 13 September where they received botulinum antitoxin.

Between 11 and 17 September 2023, 15 suspected cases of botulism among 29 exposed individuals were identified from seven countries: UK (n = 4), Canada (n =3), Ireland (n =3), the United States (US) (n =2), France (n =1), Germany (n =1) and Greece (n =1). All cases reported consuming preserved sardines marinated in oil and herbs at Restaurant A. Several cases reported a bad taste or bad smell from this dish.

**Figure 2:**
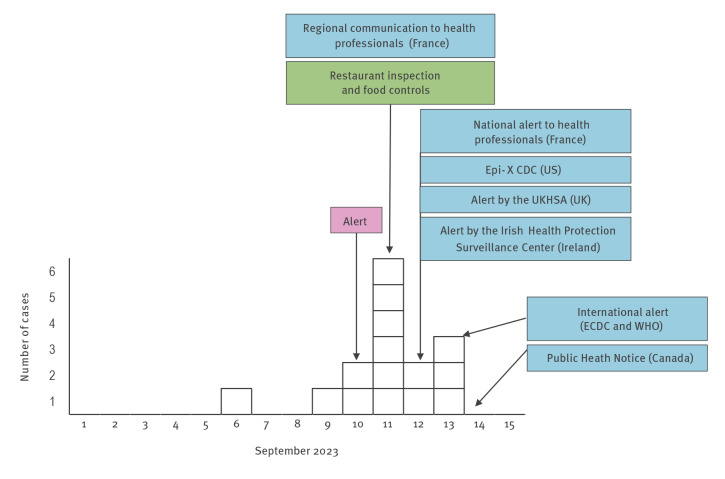
Timeline of consultation or hospitalisation of cases of botulism, outbreak control measures and communication, France, September 2023 (n = 15)

ECDC: European Centre for Disease Prevention and Control; Epi-X CDC: Epidemic Information Exchange of the Centers for Disease Control and Prevention; UK: United Kingdom; UKHSA: United Kingdom Health Security Agency; US: United States of America; WHO: World Health Organization.

The cases diseased between 5 and 12 September (median incubation period: 1 day, range 1–7) (Figure 2). One case died. The median age of the cases was 36 years (range: 30–70). Of the 15 cases, seven were female and eight male and 13 were hospitalised with six requiring invasive mechanical ventilation. All except for the deceased case received heptavalent ABCDEFG botulinum antitoxin [2].

Epi-X is a secure, web-based network for information exchange.

## Outbreak control measures

On 11–12 September, broad communication towards healthcare professionals in France, including emergency units, was carried out at regional and national levels through a dedicated health alert notification system and the general population was informed of the outbreak through regional and national press releases [3-5]. An EpiPulse message by the European Centre for Disease Prevention and Control (ECDC) was posted to inform other countries. Conference calls were organised with public health agencies of countries with affected citizens or involved in patient care (UK, Ireland, Canada, US, Germany, Spain). On 11 September, the French food safety authorities prohibited Restaurant A from storing, handling, processing, selling or delivering food products.

Simultaneously on 12 September, the UK Health Security Agency (UKHSA) issued an urgent public health alert to all frontline healthcare professionals, the Food Standards Agency and informed the public through the media. On 12–13 September, the Irish Health Protection Surveillance Centre informed relevant parties, including public health professionals, consultants in microbiology, emergency medicine and infectious diseases, the Irish Department of Health and the Irish Society of Travel Medicine, the Irish College of General Practitioners Society, the Food Safety Authority and the general public [6]. In Germany, the Robert Koch Institute informed the federal state surveillance offices and the Ministry of Health about the outbreak. The US Centers for Disease Control and Prevention (CDC), via the Epidemic Information Exchange (Epi-X) alert system, alerted the US health departments and healthcare providers on the risk of botulism among symptomatic travellers from Bordeaux. On 14 September, the Public Health Agency of Canada issued a Public Health Notice about the outbreak, online as well as on social media [7] and an international alert was issued by the World Health Organization (WHO) [8].

## Food and environmental investigation

On 11 September, the District Food Control Authority inspected the identified restaurant. Samples were taken from food items: canned sardines, marinade ingredients (garlic, cilantro, cumin seeds, sunflower oil and mustard seeds), coppa, kimchi, foie gras, eggplants, couscous, stewed lamb, dried beef and jams and sent to the National Reference Centre (NRC) for Anaerobic Bacteria and Botulism (Institut Pasteur, Paris) for laboratory analysis. The inspectors did not identify any deviations in food storage but noted incorrect sterilisation techniques in the preparation of canned food.

## Laboratory investigation

Among the 15 suspected cases, 10 were laboratory-confirmed, including seven at the NRC (serum tested positive for type B BoNT by mouse bioassay (n = 3) and/or presence of type B *C. botulinum* by real-time PCR in stools (n = 6)) and three at the UKHSA Gastrointestinal Bacteria Reference Unit (presence of type B *C. botulinum* by real-time PCR or culture in stools). Five suspected cases could not be confirmed, with either negative or inconclusive serum testing or because a stool sample could not be obtained. Among the food samples analysed, the sardine samples of five different jars tested positive for type B BoNT and type B *C. botulinum*. All other food samples including marinade ingredients tested negative for BoNT (by mouse bioassay).

## Discussion

Foodborne botulism is a serious acute and potentially life-threatening disease caused by ingestion of BoNT preformed in foods contaminated by *C. botulinum* and other BoNT-producing clostridia. Spores of these ubiquitous bacteria are dormant in the environment, but germination and toxin production in food can occur under anaerobic conditions, low acidity (pH > 4.6), low salt conditions (NaCl < 10%) and at non-refrigeration temperatures (T > 3 °C (37°F)) [9]. Nine toxinotypes of BoNT have been recognised, termed A–G, H or H/A or F/A and X; type A, B and E are primarily associated with foodborne botulism [10].

Symptoms usually appear from 12 to 36 hours up to 8 days after consumption of contaminated food, depending on the amount and type of the toxin [9,11]. The illness is characterised by an acute, afebrile, symmetric descending flaccid paralysis that can cause respiratory failure and death. Case fatality rates reported in the literature range from 3 to 10%. Early absence of physical signs of illness at examination might delay disease recognition.

Confirmation of the clinical diagnosis relies on detection of BoNT in serum, stools and suspected foods and on detection of the bacterium by culture-based or molecular methods [12,13].

This severe food poisoning requires urgent diagnosis, intensive respiratory care when needed and rapid identification of the source of the infection to prevent additional cases and effectively administer antitoxin to affected patients at an early stage.

In France, botulism is a mandatory notifiable disease with 15–20 cases reported annually, type B being the most common [14].

In this outbreak investigation, rapid identification of customers exposed to the implicated food was possible through interrogation of credit card payments and retrieval of personal contact information via the credit card companies. Given the severity of botulism, the credit card companies fully cooperated with the health authorities and contacted the identified customers for approval before forwarding the contact details. This enabled contacting and urgently referring three symptomatic British citizens unaware of their illness to an emergency unit for prompt administration of botulism antitoxin. By the time the outbreak was recognised, the majority of exposed persons had already returned to their home countries, nevertheless, all were identified via credit card companies and were provided with a public health emergency contact should symptoms occur. In addition, close collaboration between national health agencies of different countries contributed to rapid dissemination of information on risk exposure and allowed appropriate follow-up and case management.

In foodborne outbreak investigations, quickly accessible data, such as loyalty cards or purchase receipts are currently frequently used to identify food products purchased [15-18]. These methods are complementary to other methods of epidemiological investigation [19]. Because of data protection regulation, access to personal data through credit card receipts is not always feasible in a timely manner. This practice cannot be routinely applied during epidemiological investigations given the sensitivity of the data.

This botulism outbreak is unusual regarding its international dimension. During mass gatherings, such as international sport events, specific health surveillance is frequently set up as many hazards can arise such as infectious, environmental or bioterrorist-related outbreaks, including botulism [20]. Given the potential severity of the disease and the possibility of intentional use of botulism toxin as biological weapon, each case of botulism requires careful epidemiological investigation and urgent identification of the source.

We quickly considered bioterrorism as an unlikely cause of this outbreak as consumption of canned sardines from restaurant A were rapidly identified as the single commonality. Although foodborne botulism outbreaks associated with commercial products are rare in France, our findings are consistent with previous outbreaks linked to poorly sterilised foods associated with survival of *C. botulinum* spores during the food preparation process [21]. Previous reports of botulism caused by fish- and other marine products have been linked to type E BoNT [22,23]. Type B BoNT has primarily been associated with processed pork products [24].

## Conclusion

This outbreak of foodborne botulism in France highlights both the effectiveness of using credit card data to rapidly identify exposed persons and possibly prevent severe cases. It also underlines the importance of efficient international collaboration networks, particularly in mass gatherings when people from many countries can be exposed, such as in the coming Olympic Games organised in France in summer 2024.
